# Encapsulation of zinc-rifampicin complex into transferrin-conjugated silver quantum-dots improves its antimycobacterial activity and stability and facilitates drug delivery into macrophages

**DOI:** 10.1038/srep24184

**Published:** 2016-04-26

**Authors:** Rashmirekha Pati, Rojalin Sahu, Jagannath Panda, Avinash Sonawane

**Affiliations:** 1 School of Biotechnology, Campus-11, KIIT University, Bhubaneswar, Orissa-751024, India; 2 School of Applied Sciences, Campus-3, KIIT University, Bhubaneswar, Orissa-751024, India

## Abstract

In order to improve the chemotherapy of tuberculosis, there is an urgent need to enhance the efficacy of existing agents and also to develop more efficient drug delivery systems. Here, we synthesized a novel anti-TB drug complex consisting of zinc and rifampicin (Zn-RIF), and encapsulated it into transferrin-conjugated silver quantum-dots (Zn-RIF-Tf-QD) to improve delivery in macrophages. Successful synthesis of Zn-RIF and Zn-RIF-Tf-QD was confirmed by UV/Vis-spectroscopy, TEM, FTIR, photoluminescence, XRD, XPS, and NMR. The sizes of silver QDs and transferrin-conjugated QDs were found to be in the range of 5–20 nm. Activity assays showed that Zn-RIF-Tf-QD exhibited 10-fold higher antibacterial activity against *Mycobacterium smegmatis* and *Mycobacterium bovis*-BCG as compared to Zn-RIF, RIF and Zn. Immunofluorescence studies showed that Zn-RIF-Tf-QD-conjugates were actively endocytosed by macrophages and dendritic cells, but not by lung epithelial cells. Treatment with Zn-RIF-Tf-QD efficiently killed mycobacteria residing inside macrophages without exhibiting cytotoxicity and genotoxicity. Moreover, the conjugates remained stable for upto 48 h, were taken up into the late endosomal compartment of macrophages, and released the drug in a sustainable manner. Our data demonstrate that Zn-RIF-Tf-QDs have a great potential as anti-TB drugs. In addition, transferrin-conjugated QDs may constitute an effective drug delivery system for tuberculosis therapy.

Tuberculosis (TB), caused by *Mycobacterium tuberculosis* (*M. tb*), is one of the world’s major health problems. Approximately one third of the world’s population is infected with TB, resulting in 1.5 million deaths per year from this single disease alone^1^. Despite of significant advancements in the development of anti-TB drugs, the prospects of controlling this disease are worsening due to the emergence of drug resistant strains. According to a World Health Organization report there were about 480000 new cases of multidrug-resistant TB (MDR-TB) and 43,200 cases of extensively drug-resistant TB (XDR-TB) worldwide^2^. Strains that are resistant to at least rifampicin (RIF) and isoniazid (INH) (the two most effective first line drugs used to treat TB) are classified as MDR-TB, whereas XDR-TB strains are resistant to any of the fluoroquinolones, and to at least one of the three injectable second-line drugs (capreomycin, kanamycin, and amikacin), in addition to INH and RIF^3^. Using these drugs, resistant levels and frequent treatment failures are major concerns. Although a large number of novel drugs have been discovered in the past few decades, many of them have failed in clinical trials due to their adverse effects and/or their inefficacy as therapeutic molecules.

The current treatment of TB patients is based on the principle of combination chemotherapy. The “first line” therapy consists of three or four drugs i.e. isoniazid, rifampicin, pyrazinamide and ethambutol, followed by the less efficacious and more expensive “second line” drugs, which include capreomycin, kanamycin, amikacin, para-aminosalicylic acid, ciprofloxacin, ofloxacin, moxifloxacin, and clofazimine. Major limitations associated with the current TB therapy include prolonged treatment duration and the use of high doses of antibiotics, which result in severe adverse effects and non-compliance of the patients. These factors are mainly due to the prevalence of drug resistant bacterial strains. Furthermore, mycobacteria have developed various strategies to survive and replicate inside macrophages for extended periods of time. These strategies include inhibition of phago-lysosome fusion, phagosomal escape, manipulation of phagosomal vesicle environment, and inhibition of oxidative stress responses^4^,^5^,^6^,^7^. To overcome these challenges, it is imperative to improve the anti-TB activity of existing drugs by combining them with other antibacterial molecules that have different modes of action and/or synergistic activity. Moreover, as *M. tb* is an intracellular pathogen, it is important to deliver these drugs specifically to the macrophage compartment where *M. tb* resides.

Metal ions have many important physiological functions in the body. Transition metal complexes exhibit unique and interesting properties such as changing oxidation states and the ability to form specific interactions with other biomolecules^8^,^9^. Thus transition metals and their complexes have been used for the development of drugs with promising pharmacological applications and may offer unique therapeutic opportunities^10^. It was shown that some metal-drug complexes are more potent as compared to pure drug^11^,^12^,^13^. Toward this end, the interactions of some antibiotics with transition metals have been studied^14^. Among them, zinc is known to exhibit antibacterial activity^15^. Zinc oxide (ZnO) nanoparticles are even more efficient along with reduced toxicity^16^.

Rifampicin (RIF), a broad-spectrum antibiotic, is one of the most effective first line drugs against TB. RIF binds to the β-subunit of RNA polymerase and thus inhibits the elongation of messenger RNA^17^. At therapeutic levels, RIF exhibits a proven bactericidal activity against *M. tb*. Daily doses of about 600 mg are usually administered, which yield serum drug concentrations well above the minimum inhibitory concentration of RIF against *M. tb* but are also associated with hazardous side effects such as hepatotoxicity, antibody formation, fever, thrombocytopenia and renal failure^18^,^19^.

Semiconductor nanocrystals, also known as quantum dots (QDs), have become an important tool in biomedical research, especially for quantitative and long-term fluorescence imaging and detection^20^,^21^,^22^,^23^. Engineered QDs with integrated targeting, imaging and therapeutic functionalities have also become excellent materials for drug delivery into cells and whole animals. Because of their high drug loading capacity and their ability to cross various barriers, QDs have been used for the intracellular delivery of different types of peptides, drugs and nucleic acids^24^,^25^.

The major objective of this work was to develop a novel drug composite together with an efficient drug delivery system to improve TB therapy. In view of this goal, we synthesized a zinc-rifampicin (Zn-RIF) complex and checked its activity against non-pathogenic *Mycobacterium smegmatis*, and *Mycobacterium bovis*-BCG, which behave like pathogenic *M. tb*. We chose RIF because it is known to coordinate with metal cations through various chemical groups i.e. two phenolic and two aliphatic OH groups. Moreover, its flexible backbone together with the presence of additional nitrogen and oxygen donor atoms render this compound interesting for studying its coordination behavior with transition metal ions.

Further to improve drug delivery inside the macrophages, we encapsulated the Zn-RIF complex in transferrin-conjugated QDs and investigated its *in-vitro* and *ex-vivo* antimycobacterial activity, targeting properties and intracellular stability in various primary immune cells isolated from mice. Synthesis of Zn-RIF complex and its conjugation to transferrin coupled QDs was studied by UV-Visible spectroscopy, transmission electron microscopy (TEM), Fourier transform infrared spectroscopy (FTIR), photoluminescence, X-ray diffraction (XRD), X-ray photoelectron spectroscopy (XPS), and Nuclear magnetic resonance (NMR). We observed that conjugation of Zn-RIF complex to transferrin coupled QDs exhibit significantly higher antimycobacterial activity under both *in-vitro* and *ex-vivo* conditions when compared with the Zn-RIF complex, and RIF or Zn alone. These drug encapsulated QDs were efficiently internalized by mycobacteria primary host cells such as macrophages and dendritic cells and exhibited slow drug release behavior as evident from the intracellular stability assay. Further, we showed that these drug loaded QDs were localized in the late endosomal compartment and exhibited no symptoms of cytotoxicity and genotoxicity on the host cells.

## Results

### Characterization of the Zn-RIF complex

#### Fourier transform infrared spectroscopy (FTIR)

**Figure 1:**
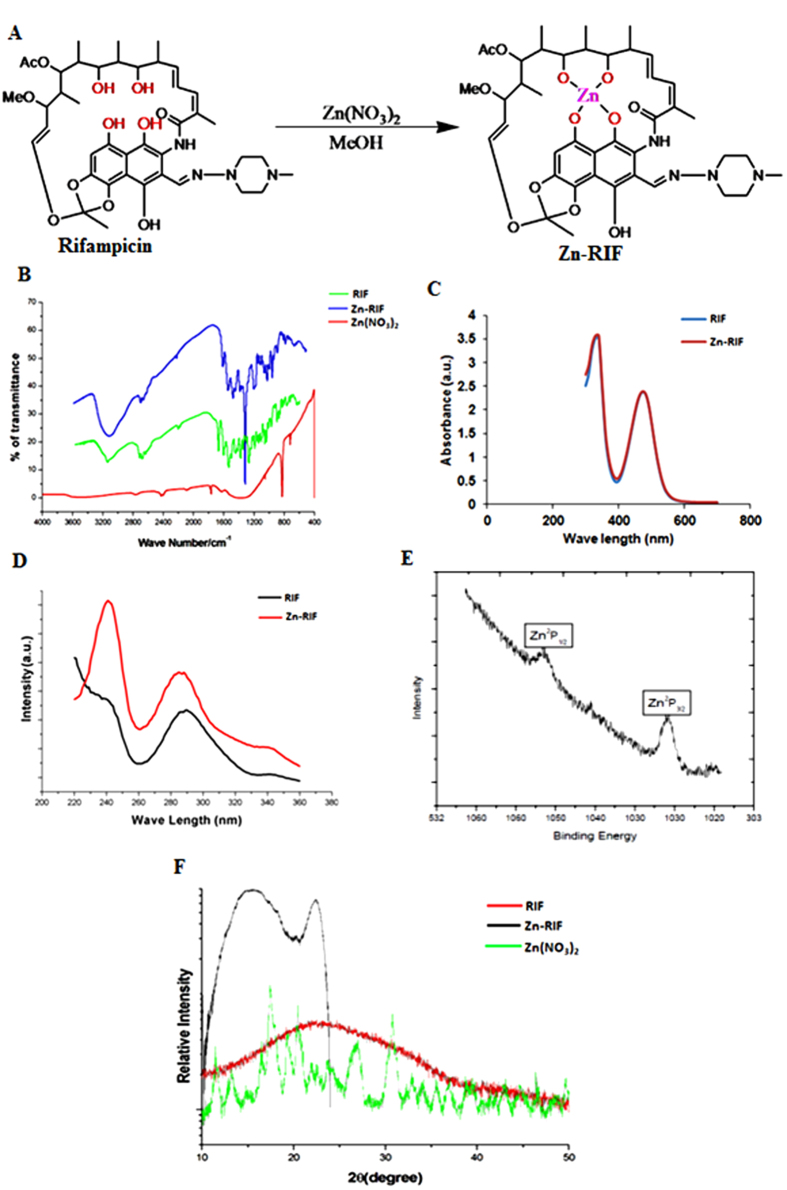
Synthesis and Characterization of Zn-RIF Complex. (**A**) Synthesis of Zinc and rifampicin complex. **(B)** FTIR spectrum of Rifampicin (green line), Zn-RIF complex (blue line) and Zn(NO_3_)_2_ (red line); **(C)** UV-Vis spectrum of Rifampicin (blue line) and Zn-RIF complex (red line); **(D)** Photoluminescence spectrum of Rifampicin (black line) and Zn-RIF complex (red line); **(E)** XPS analysis of Zn-RIF complex; **(F)** Powder XRD (P-XRD) analysis of Rifampicin (red line), Zn-RIF complex (black line) and Zn(NO_3_)_2_ (green line).

The structure of the Zn-RIF complex (Fig. 1A) was confirmed by several physico-chemical methods. The IR spectrum of Zn-RIF was compared with that of free RIF (Fig. 1B). In agreement with a previous report^26^, the spectrum of free RIF showed characteristic peaks at 3482 (O-H), 2880,1726 (C=O), 1646(C=N), 1247(COC) and 808(C-H) cm^−1^. A characteristic broad band at 3474 cm^−1^ was observed with the Zn-RIF (Fig. 1B), which was absent in free RIF. This band may correspond to the stretching of phenolic and aliphatic free OH groups of RIF. Complex formation of RIF with Zn shifted ν(O-H) to a lower frequency and the band became more enlarged in that region (3453 cm^−1^). These results indicate that OH groups participate in the coordination of RIF to Zn.

#### UV-Vis Spectra analysis of the Zn-RIF complex

UV-Vis spectrometry analysis did not show any difference in the spectra of RIF and Zn-RIF complex (Fig. 1C). The Zn-RIF complex exhibited only charge transfer transitions, from the ligand (RIF) to the metal and *vice versa*. Therefore, no d-d transitions are expected for d^10^Zn(II) complexes^27^. Both RIF and Zn-RIF showed strong absorption bands in the range 390–420 nm (Fig. 1C), which may be associated with a π→π* and n→π* transitions originating mainly in the RIF chromophore (carbonyl, double bonds, imine, n→π*transition).

#### Photoluminescence analysis of the Zn-RIF complex

We also performed photoluminescence experiments to confirm the formation of the Zn-RIF complex. The photoluminescence properties of the complex, which is a d^10^ system, has been used in various biological applications such as in chemical sensors, photochemistry and inorganic LEDs^28^. The emission spectra of free RIF and the Zn-RIF complex at λ_ex_ = 280 nm are depicted in Fig. 1D. It can be seen that the free RIF exhibited a much weaker emission band as compared to Zn-RIF (Fig. 1D). This could be due to an intra-ligand emission state, which is characteristic of fluorescent emission spectra of Zn complexes^29^.

#### X-ray photoelectron spectroscopy

The oxidation state of Zn was confirmed by high-resolution X-ray photoelectron spectroscopy (XPS). From the peak positions of the elements present in the sample their binding energies can be determined. As shown in Fig. 1E, the XPS spectrum of Zn showed two peaks corresponding to Zn 2p_3/2_ at 1030 eV and to Zn 2p_1/2_ at 1045 eV. The Zn 2p_3/2_ peak is much higher than the Zn 2p_1/2_ peak indicating the involvement of Zn^2+^ in complex formation^30^ (Fig. 1E).

#### X-ray powder diffraction analysis of the Zn-RIF complex

X-ray diffraction (XRD) analysis of Zn-RIF was carried out using CuKα at *λ* = 1.54 Å and Θ-2θ scans from 20° to 80° with a scan speed of 0.5°/min and an incidence angle of 1° at a step size of 0.05°. The powder XRD spectra of the Zn-RIF, free RIF and Zn(NO_3_)_2_ shown in Fig. 1F confirm the formation of the Zn-RIF complex. RIF is amorphous in nature, which reduces peak intensity. However, RIF complexed with Zn produces a sharp peak at an angle of 20°–25°. Another intense peak at angle of 15° showed the presence of the ligand i.e. RIF^31^.

#### NMR spectroscopy

**Figure 2:**
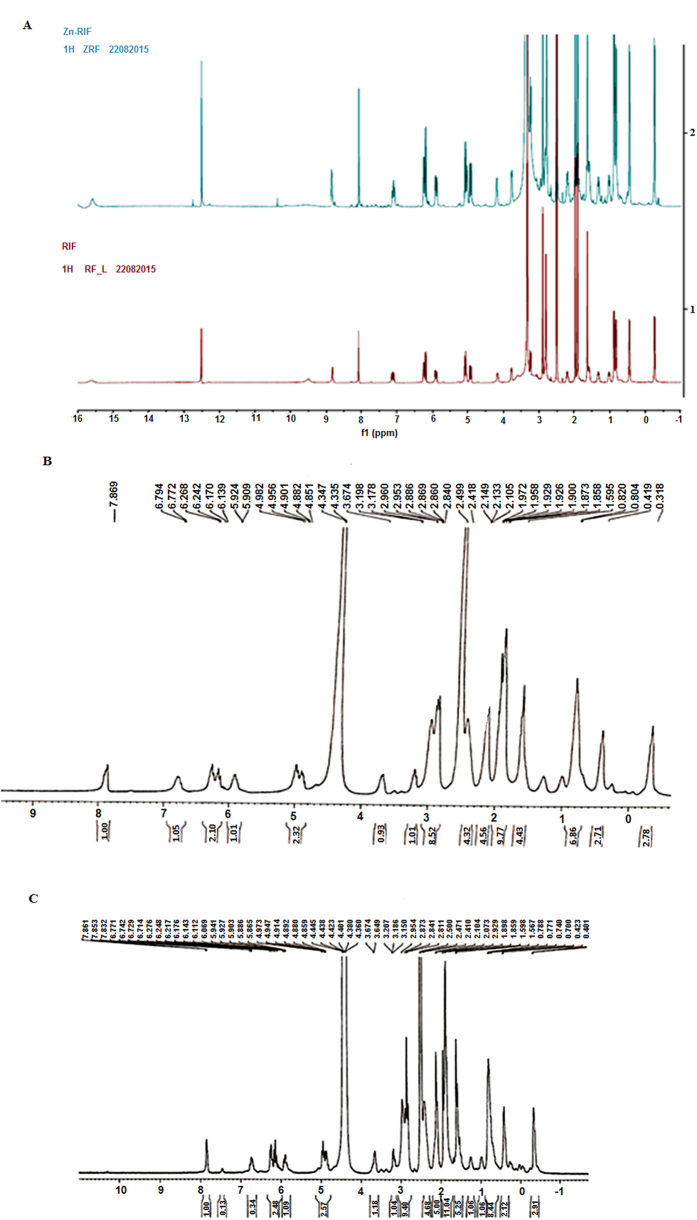
Characterization of Zn-RIF complex. (**A)** NMR Spectrum of Zn-RIF complex (blue line) and Rifampicin (red line);Deuterium exchange in **(B)** Rifampicin and **(C)** Zn-RIF complex.

^1^H NMR spectrum analysis of RIF and the Zn-RIF complex was carried out in DMSO-D_6_ using a Bruker AVANCE 400 NMR spectrometer. Tetramethylsilane (TMS) was used as an internal standard. All peaks arising from unbound RIF ligand were clearly resolved (Fig. 2A) with the phenolic hydroxyl protons appearing at 9–10 (2 H) ppm. In the Zn-RIF spectrum these peaks were absent due to replacement with zinc. To detect exchangeable protons, we performed deuterium exchange experiments with D_2_O. It was observed that all the protons with shifts between 8 and 16 ppm were exchanged with deuterium (Fig. 2C). By comparing the splitting patterns, it can be concluded that the metal to ligand ratio is 1:1^32^.

### The Zn-RIF complex showed enhanced antimycobacterial activity as compared to free RIF and zinc

**Figure 3:**
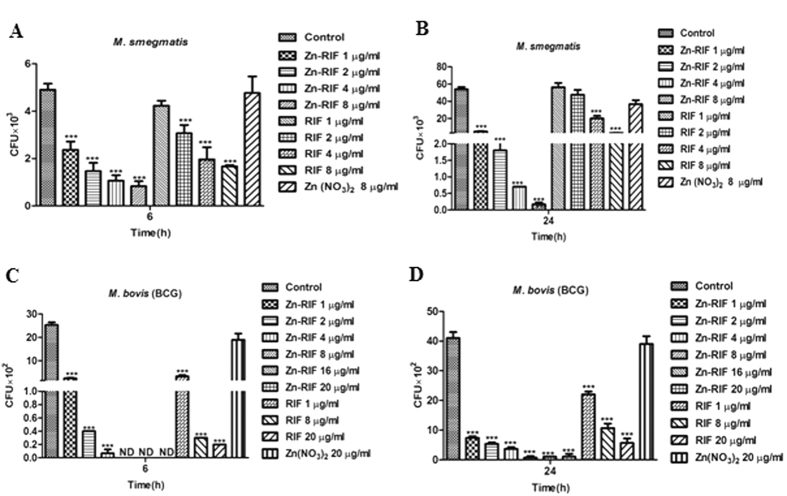
Antibacterial activity of Zn-RIF complex against *M. smegmatis* at **(A)** 6 h, **(B)** 24 h and *M. bovis-BCG* at **(C)** 6 h and **(D)** 24 h. Bacteria were incubated with different concentrations of Zn, RIF and Zn-RIF complex for the indicated time points. Bacterial survival was determined by CFU assay. Experiments were performed in triplicates, results are shown mean ± SD; ***P ≤ 0.001.

We compared the antibacterial activity of Zn-RIF complex against *M. smegmatis* and *M. bovis*-BCG with Zn and RIF alone. *M. smegmatis* is a non-pathogenic mycobacterial strain, whereas *M. bovis*-BCG behaves like pathogenic mycobacteria. Exponentially grown bacteria were incubated with various concentrations of RIF, Zn and Zn-RIF, and surviving colonies were counted after 72 h for *M. smegmatis* and 3 weeks for *M. bovis-BCG*. Free RIF and free Zn in concentrations corresponding to those in Zn-RIF were used as controls. The results showed distinct differences in susceptibility of mycobacteria. As shown in Fig. 3, Zn-RIF exhibited significantly higher antibacterial activity in a dose-dependent manner when compared with Zn and RIF alone. After 6 h of incubation, approximately 53% and 83% (P ≤ 0.01 and P ≤ 0.001) of the *M. smegmatis* population was killed at 1 and 8 μg/ml concentrations of Zn-RIF, respectively; whereas after 24 h of incubation at similar concentrations about 91% and 99% (P ≤ 0.001) of *M. smegmatis* were killed (Fig. 3A,B). With RIF alone, only 14.2 and 67.5% killing was observed at 1 and 8 μg/ml after an 6 h incubation; whereas no bacterial killing at all was observed after exposure to Zn(NO_3_)_2_ at equimolar concentrations. Similarly, after 24 h of incubation, RIF and Zn(NO3)_2_ at 1 μg/ml concentration showed no killing activity against *M. smegmatis*, whereas an exposure to 8 μg/ml RIF inhibited 94% of bacterial growth (Fig. 3A,B). In comparison to *M. smegmatis, M. bovis-*BCG was more susceptible to Zn-RIF action such that exposure to 1 μg/ml of Zn-RIF complex killed approximately 90% (Fig. 3C, P ≤ 0.001) of *M. bovis*-BCG, whereas no colonies at all were observed at 8 μg/ml after 6 h of incubation. After 24 h exposure to Zn-RIF at 1 and 8 μg/ml, approximately 82% and 98% of *M. bovis*-BCG cells were killed (Fig. 3D; P ≤ 0.001). RIF alone at 1 and 8 μg/ml killed 46% and 86% of *M. bovis*-BCG cells, whereas Zn(NO_3_)_2_ alone had no effect after 24 h of treatment. These data indicate that Zn-RIF exhibits significantly higher antimycobacterial activity as compared to RIF and Zn(NO_3_)_2_ alone.

### Zn-RIF kill mycobacteria residing inside macrophages

**Figure 4:**
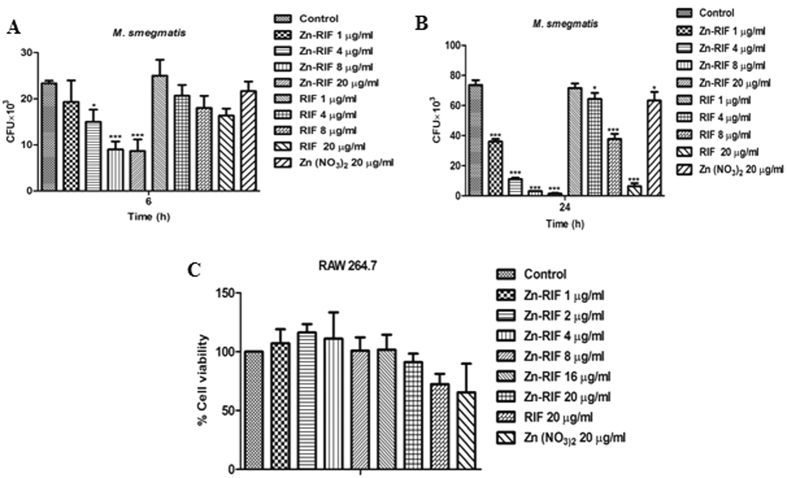
Zn-RIF complex exhibit intracellular killing activity and no cytotoxicity on macrophages. RAW 264.7 cells were treated with different concentrations of Zn-RIF complex, RIF and Zn(NO_3_) 1 h after (post treatment) *M. smegmatis* infection. Macrophages infected with bacteria alone were used as control. The cells were lysed at **(A)** 6 h and **(B)** 24 h post infection and the intracellular bacterial survival was determined by CFU assay. **(C).** Cytotoxic activity on RAW 264.7 cells. Cells were treated with different concentrations of RIF, Zn(NO_3_) and Zn-RIF complex for 24 h. Cell viability was determined by MTT assay. Experiments were performed in triplicates, results are shown mean ± SD; *P ≤ 0.1; ***P ≤ 0.001.

Fighting intracellular pathogens is a major challenge as the drugs used must enter the cells to kill the bacteria. Since mycobacterium is an intracellular pathogen^33^, we checked the intracellular burden of *M. smegmatis* after treatment of macrophages with RIF, Zn and Zn-RIF. As shown in Fig. 4A, 6 h treatment with Zn-RIF at 8 and 20 μg/ml resulted in an approximately 3-fold decrease of the intracellular bacterial burden as compared to RIF and Zn alone. After an 24 h treatment with 1 and 20 μg/ml, the intracellular burden of *M. smegmatis* was reduced by 50% and 98%, respectively; whereas RIF alone killed 2.7% and 91% of bacteria under similar conditions (Fig. 4B).

### Zn-RIF in bactericidal doses did not exert cytotoxicity on mouse macrophages

From the therapeutic perspective, it is important that drugs should kill intracellular pathogens without exhibiting cytotoxic effects on the host cells. In the present study, we compared the cytotoxic effects of Zn-RIF on mouse macrophages with those of RIF and Zn using the MTT assay. As shown in Fig. 4C, Zn-RIF had no cytotoxic effects on the macrophages at bactericidal concentrations, indicating that decrease in intracellular bacterial burden is not due to reduction in macrophage cell viability.

### Characterization of Zn-RIF encapsulated in transferrin-conjugated quantum dots

**Figure 5:**
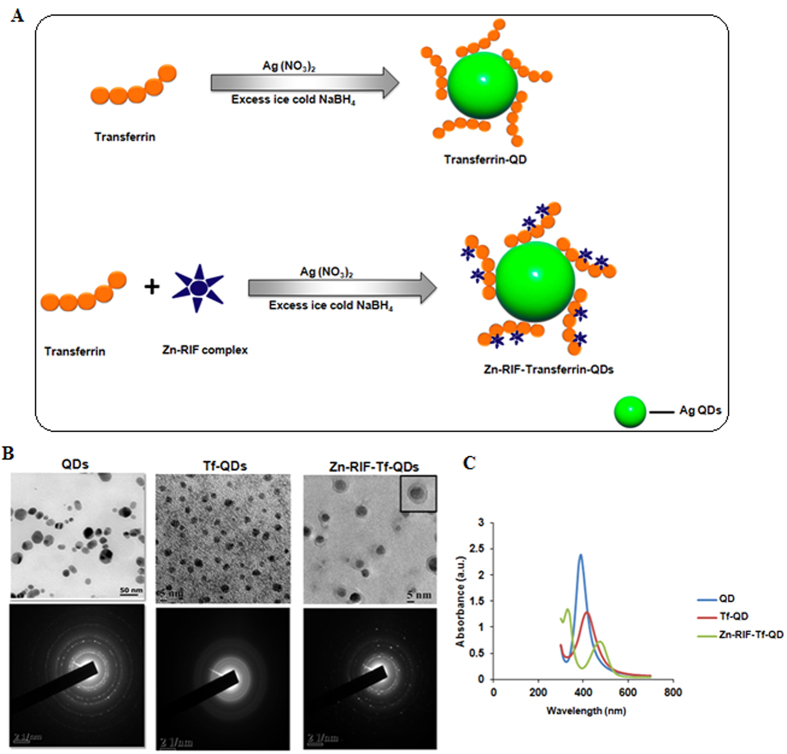
Characterization of Zn-RIF-Tf-QD. (**A)** Schematic illustration of transferrin embedded and Zn-RIF complex encapsulated transferrin embedded silver QDs. TEM image of **(B)** Ag QDs, Tf-QDs and Zn-RIF-Tf-QD. **(C)**Uv-visible absorption spectrum of QD, Tf-QD and Zn-RIF-Tf-QDs.

As mentioned above, mycobacteria are intracellular pathogens. To improve the delivery of Zn-RIF into macrophages, we encapsulated Zn-RIF in QDs that was used previously as vehicles for drug delivery to various cell type^34^. To further increase the specificity of the system for macrophages, we conjugated the QDs to transferrin (Tf). The scheme for the synthesis of Zn-RIF-Tf-QD conjugates is shown in Fig. 5A. The resultant Zn-RIF-Tf-QD conjugates were characterized by various physico-chemical methods.

#### Transmission electron microscopy

The size and morphology of silver QDs, transferrin conjugated QDs (Tf-QDs) and Zn-RIF encapsulated Tf-QDs (Zn-RIF-Tf-QDs) were characterized using TEM. As shown in Fig. 5B (upper panel), mono-dispersed spherical Zn-RIF-Tf-QD nanoparticles were obtained with a mean diameter of 4.0 to 0.6 nm. The TEM analysis also confirmed the conjugation of transferrin to silver QDs. The size of Tf-QDs was about 5 nm, whereas silver QDs had diameters of about 20 nm. This decrease in size upon Tf-conjugation could be due to the fact that proteins act as a stabilizer and it also help in reducing the size of the particles and prevent their aggregation. However, encapsulation of Zn-RIF in Tf-QDs again increased their size. TEM images of Zn-RIF-Tf-QD conjugates showed the presence of a layer surrounding the QDs (Fig. 5B, right upper panel). We speculate that transferrin first bind to QDs to form a complex (Tf-QDs), which then facilitates the binding of Zn-RIF by covalent and non-covalent interactions (Fig. 5B). High resolution TEM images showed the presence of lattice fringes in Zn-RIF-Tf-QD conjugates (Fig. 5B, lower panel), indicating the formation of a nano crystalline material. Altogether, the above data confirm the formation of Zn-RIF complex and the conjugation of Tf with QDs.

#### UV-Vis Spectral analysis

The Zn-RIF-Tf-QD sols were yellow and showed a single visible band near 400 nm (Fig. 5C), which is characteristic of silver QDs^35^. The sols were stable for several weeks at room temperature with no precipitation or change in color on standing. The Tf-QD complex showed a bathochromic shift with a maximum peak at around 450 nm. Moreover in comparison to QDs, the peak intensity of Tf-QD also decreased due the binding of transferrin. The Zn-RIF-Tf-QD exhibited two bands at 320–340 nm and 460–480 nm, which originate from QDs. This band was broadened due to interaction with the transferrin molecule (Fig. 5C). With increasing Zn-RIF and transferrin precursor ratio the absorption spectra and emission spectra of the Zn-RIF-Tf-QD was shifted towards longer wavelength (Fig. 5C).

### Zn-RIF encapsulated transferrin-conjugated QDs (Zn-RIF-Tf-QD) had higher *in-vitro* bactericidal activity as compared to Zn-RIF

**Figure 6:**
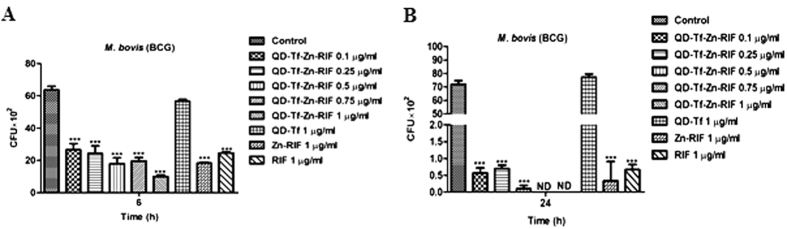
Antibacterial and cytotoxic activity of Zn-Rif-Tf-QDs. Dose dependent killing of *M. bovis*-BCG by Zn-RIF, Tf-QDs, RIF and Zn-RIF-Tf-QD after **(A)** 6 h and **(B)** 24 h. Bacteria were incubated with different concentrations of Zn-RIF, Tf-QDs, RIF and Zn-RIF-Tf-QD and their survival was determined at the indicated time points by CFU assay. Media containing bacteria alone was used as control and the corresponding concentrations of RIF and Zn(NO_3_) present in respective doses of Zn-RIF complex were used as rifampicin and Zn(NO_3_) controls, respectively. Experiments were performed in triplicates, results are shown mean ± SD; ***P ≤ 0.001.

First, we compared the antibacterial activity against *M. bovis*-BCG of Zn-RIF-Tf-QD with those of RIF and Zn-RIF under *in vitro* condition. As shown in Fig. 6A, exposure of *M. bovis-*BCG to 0.1 and 1 μg/ml of Zn-RIF-Tf-QD for 6 h inhibited growth by 58% and 84%, respectively, whereas no bacterial killing was observed by RIF and Zn-RIF at 0.1 μg/ml . Exposure to 1 μg/ml of RIF and Zn-RIF inhibited *M. bovis-*BCG growth by 61% and 71%, respectively. After 24 h, we observed an approximately 10-fold higher killing rate by Zn-RIF-Tf-QD as compared to RIF and Zn-RIF (Fig. 6B). Treatment with 0.1 μg/ml Zn-RIF-Tf-QD killed 99% of the bacteria, which is equivalent to the killing observed after exposure to 1 μg/ml concentration of RIF and Zn-RIF (Fig. 6B). No bacterial colonies were observed at 0.75 μg/ml and higher concentration of Zn-RIF-Tf-QD (Fig. 6B). These data indicate that Zn-RIF-Tf-QD are at least 10-fold more potent than RIF and Zn-RIF.

### Zn-RIF-Tf-QDs are more efficient in killing intracellular mycobacteria in macrophages

**Figure 7:**
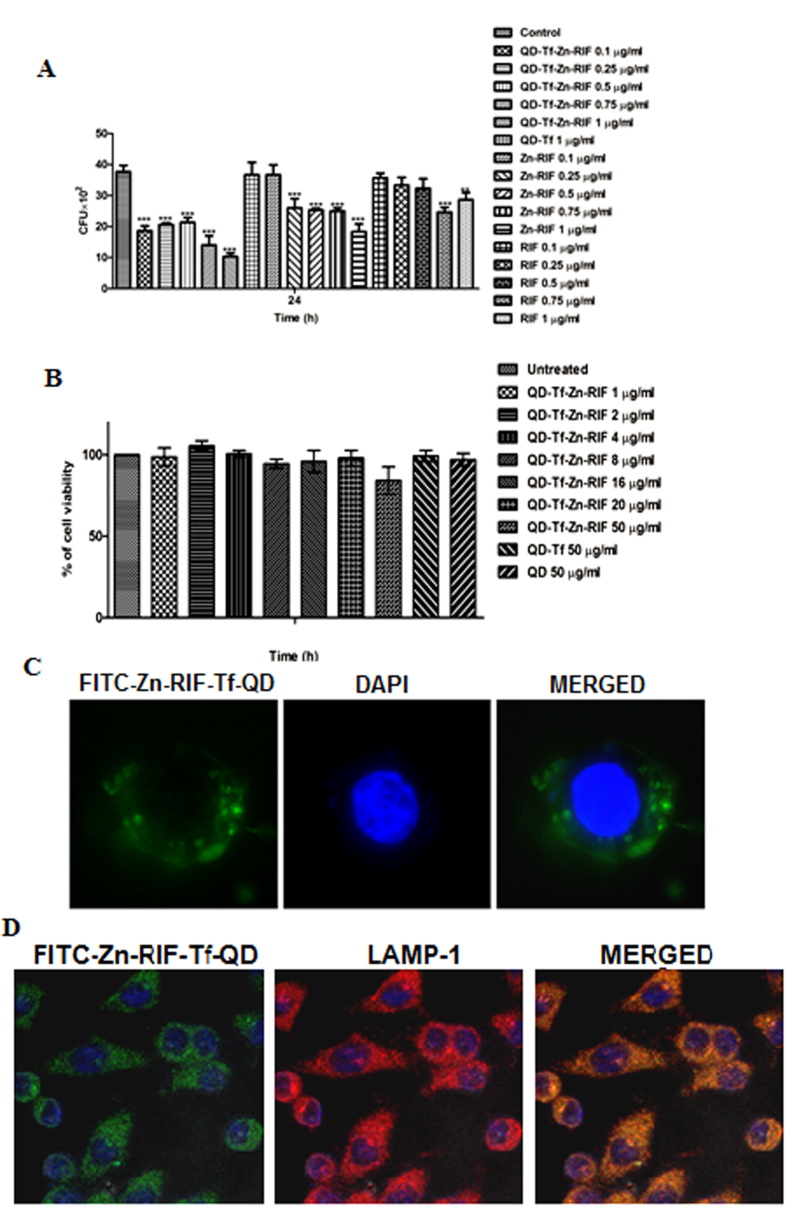
Intracellular killing activity, cytotoxicity and cellular endocytosis of Zn-RIF-Tf-QDs. (**A)** Zn-RIF-Tf-QDs exhibit intracellular killing activity against *M. bovis*-BCG. RAW 264.7 cells were incubated with different concentrations of RIF, Zn-RIF, QD-Tf and Zn-RIF-Tf-QDs 2 h after *M. bovis*-BCG infection. Macrophages infected with bacteria alone were used as control. The cells were lysed after 24 h and the intracellular bacterial survival was determined by CFU assay. **(B)** Cytotoxic activity of Tf-QDs and Zn-RIF-Tf-QDs on RAW 264.7 cells. Cells were treated with different concentrations of Tf-QDs and Zn-RIF-Tf-QDs for 24 h and cell viability was determined by MTT assay. **(C)** Endocytosis of FITC-labelled Zn-RIF-Tf-QDs in RAW 264.7 cells was studied using fluorescence microscopy. **(D)** Localization of FITC-labeled Zn-RIF-Tf-QDs in macrophages was determined by immunofluoroscence. Cells were treated with FITC-labeled Zn-RIF-Tf-QDs for 3 h and the cells were stained with LAMP 1 antibody (1:1000) followed by Alexa flour secondary antibody (1:2000). Experiments were performed in triplicates, results are shown mean ± SD; **P ≤ 0.01; ***P ≤ 0.001.

Next we compared the intracellular bacterial killing efficacy of Zn-RIF-Tf-QD with those of Zn-RIF and RIF in macrophages infected by *M. bovis* BCG. *M. bovis*-BCG resides in the phagosomal compartment of macrophages. We hypothesized that the presence of Tf on QDs should facilitate the delivery of Tf-containing drug complex to phagosomes through interaction with the Tf-receptor, thereby causing more efficient bacterial killing. To test this assumption, RAW 264.7 cells were treated with 0.1, 0.25, 0.5, 0.75 and 1 μg/ml of Zn-RIF-Tf-QD, Zn-RIF and RIF 2 h after infection *by M. bovis* and intracellular bacterial survival was checked. As shown in Fig. 7A, significantly higher intracellular killing of *M. bovis*-BCG was observed in macrophages treated with Zn-RIF-Tf-QD as compared to untreated, and macrophages treated with Zn-RIF and RIF. Importantly, Zn-RIF-Tf-QD did not exhibit cytotoxic effects on macrophages at bactericidal doses (Fig. 7B).

### Zn-RIF-Tf-QD is actively endocytosed and localized in the late endosomal compartment of macrophages

Next, we performed fluorescence microscopic studies to examine the internalization of Zn-RIF-Tf-QD by macrophages. Since particles of a certain size can be engulfed by macrophages^36^, Zn-RIF-Tf-QDs were labeled with FITC and then added to mouse macrophages. Our microscopic studies showed active endocytosis of FITC labeled Zn-RIF-Tf-QD by macrophages (Fig. 7C).

We further checked the intracellular localization of FITC labeled Zn-RIF-Tf-QD using LAMP-1, a membrane marker of late endosome/lysozome. Indeed, Zn-RIF-Tf-QD co-localized with LAMP-1 (Fig. 7D).

### Intracellular stability of Zn-RIF-Tf-QDs in mouse macrophages and dendritic cells

**Figure 8:**
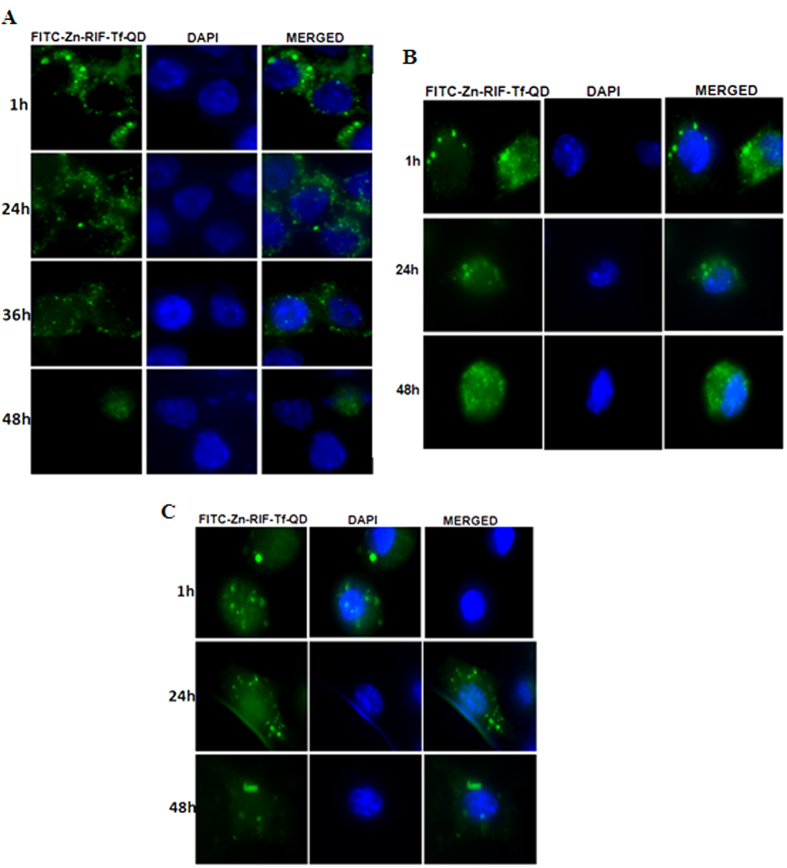
Intracellular stability of Zn-RIF-Tf-QDs complex in **(A)** RAW264.7 macrophages, **(B)** mice peritoneal macrophages and **(C)** dendritic cells.

Next, we studied the intracellular stability of FITC labeled Zn-RIF-Tf-QD in RAW264.7 macrophages, dendritic cells and peritoneal macrophages isolated from mice. Our microscopic data showed that the complex remained stable up to 48 h in all cells (Fig. 8). Afterwards a gradual decrease of fluorescence intensity was observed, indicating that Zn-RIF-Tf-QDs are quite stable inside the cells.

### Zn-RIF-Tf-QDs are selectively internalized by macrophages, but not by lung epithelial cells

**Figure 9:**
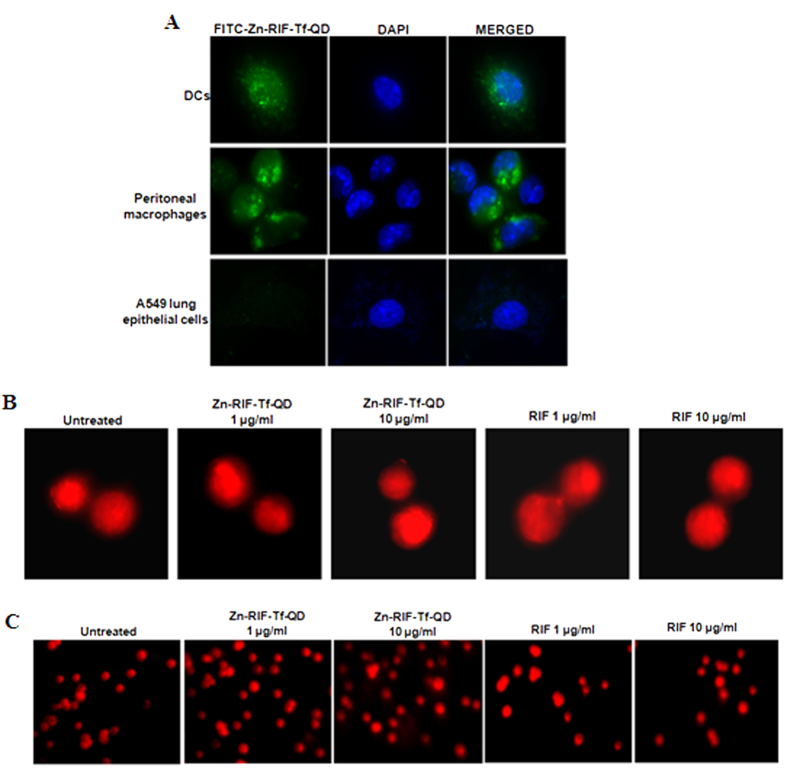
Transferrin conjugated Quantum dots are actively internalized by peritoneal macrophages and dendritic cells but not by epithelial cells and showed no genotoxicity. Uptake of FITC labeled Zn-RIF-Tf-QDs by (**A**) peritoneal macrophages, dendritic cells and lung epithelial cells. (**B,C**) Determination of genomic instability in Zn-RIF-Tf-QDs treated macrophages. RAW 264.7 cells were treated with indicated concentrations of Zn-RIF-Tf-QDs for 24 h. Untreated cells were used as control. DNA damage was determined by (**B**) Micronuclei assay and (**C**) Comet assay.

We further compared the rates of uptake of FITC labeled Zn-RIF-Tf-QD by A549 lung epithelial, peritoneal macrophages and dendritic cells. As shown in the Fig. 9A, FITC-labeled QDs were actively internalized by peritoneal macrophages and dendritic cells, but not by A549 lung epithelial cells. These results indicate that Zn-RIF-Tf-QDs are indeed specifically delivered to the macrophages.

We also examined the genotoxicity of Zn-RIF-Tf-QDs against mouse macrophages using the micronuclei and comet assays. As shown in the Fig. 9B,C, no signs of micronucleated cells or comet tail formation were observed in macrophages treated with RIF and Zn-RIF-Tf-QD.

## Discussion

The development of drug resistance and detrimental side effects of the administered drugs are of serious concerns in the treatment of many diseases. The current treatment regimen of tuberculosis is associated with multiple problems such as prolonged treatment duration, poor permeability of target cells for the drug with unpleasant side effects, difficulties in maintaining sufficiently high drug concentrations at the infected site, and premature degradation of the drug before it reaches the target site. Due to these circumstances, new drugs and novel drug delivery strategies are required to improve the TB therapy. Among them, one strategy aims at the development of new anti-TB drugs by re-engineering existing drugs to improve their antimycobacterial activity and bioavailability and reduced toxicity.

Transition metals can exhibit a wide variety of coordination properties, and reactivities, which can be used to form complex with drugs as ligands. Previously, several studies have reported improved therapeutic properties of several metal complexes against *M. tb*. RIF, which is considered the cornerstone in the short-course TB treatment regimen, exhibits detrimental side effects. To address these issues, we employed a strategy in which Zn was complexed with RIF to form a Zn-RIF complex, which was subsequently encapsulated in transferrin-conjugated silver QDs to yield the Zn-RIF-Tf-QD conjugate. Detailed physico-chemical analyses confirmed the formation and encapsulation of Zn-RIF complex in transferrin-coupled QDs. We demonstrated that encapsulation of transferrin on the surface of quantum dots enhanced the binding efficiency of drug molecules. Then we showed that transferrin-conjugated and Zn-RIF encapsulated silver QDs successfully targeted to the macrophages, remaining stable for up to 48 h and significantly reducing the bacterial burden inside the macrophages.

A major factor that contributes to the drug resistance of mycobacteria is the presence of a multi-layered hydrophobic cell wall^37^, which reduces the permeability to anti-TB drugs. Therefore, it is of utmost importance to identify molecules that act directly on the cell wall of mycobacteria. First, we evaluated the antibacterial activity of Zn-RIF against *M. smegmatis* and *M. bovis*-BCG and compared it with that of Zn and RIF alone. In this way we confirmed that Zn-RIF exhibited more potent antibacterial activity against both mycobacterial strains than Zn or RIF. This may be due to better penetration of Zn-RIF through the bacterial membrane as the complex is more lipophilic than free RIF. Moreover, Zinc nitrate is known to disintegrate bacterial cell membranes^16^. This may have facilitated the diffusion of RIF into the bacterial cell and inhibit RNA synthesis.

Efficient clearance of intracellular mycobacteria is a major challenge. Mycobacteria are intracellular pathogens that can reside inside macrophages for extended periods of time. We therefore examined the intracellular killing efficacy of Zn-RIF in mouse macrophages infected with *M. smegmatis*. We found that Zn-RIF significantly reduced intracellular bacterial burden after penetrating into the macrophages.

Despite of the widespread use of RIF in the treatment of TB, toxic effects have been reported by many researchers^38^. RIF is known to induce hepatotoxicity and lipid peroxidation in the liver and bone marrow^39^,^40^. Here, we compared the cytotoxic effects of Zn-RIF with RIF and Zn(NO_3_)_2_ on mouse macrophages by MTT assay. In our previous studies, we have shown that zinc oxide nanoparticles kill intracellular mycobacteria without causing any toxic effect on macrophages^16^. In the present study, we also observed that Zn-RIF showed less cytotoxic effects against mouse macrophage, and that no cytotoxic effects were observed at bactericidal levels. These results indicate that Zn-RIF is a promising candidate for therapeutic applications.

Quantum dots (QDs) are nano scale semiconductor crystals with sizes of 1–10 nm. QDs provide excellent tools for sensing, imaging, drug delivery and therapy due to their optical properties, broad excitation range, well-defined emission wavelengths and their ability to attain different shapes thus providing an excellent structure for coating with various biomolecules^24^,^25^,^34^,^41^. To improve the delivery of the Zn-RIF inside the macrophages, we encapsulated it in transferrin-conjugated silver QDs. As macrophages express a Tf-receptor on their surface, we hypothesized that transferrin should facilitate the uptake of drugs by the macrophages through interactions with receptor^42^,^43^. Indeed, we found that the Zn-RIF-Tf-QD exhibits at least 10-fold higher *in vitro* and intracellular killing activity as compared to Zn-RIF and free RIF, indicating that the conjugate formation leads to enhanced bactericidal efficacy. This could be due to the targeted delivery of the conjugate to the mycobacteria-containing phagosomes followed by the slow release of Zn-RIF from the conjugate.

It is now well established that pathogenic mycobacteria survive inside the macrophages by preventing the phago-lysosome fusion. Therefore, from the therapeutic perspective it is important that drug molecules be trafficked via the endocytic pathway to achieve direct killing effects on mycobacteria. By fluorescence microscopy and immunofluoroscence staining we confirmed that FITC labeled Zn-RIF-Tf-QDs were indeed endocytosed by the macrophages and eventually co-localize with LAMP-1, indicating that that the drug composite taking the endocytic pathway of macrophages.

Many drugs are either prematurely degraded or pumped out from their target cells through efflux pumps, which impair their therapeutic efficacy. Therefore, in the therapeutic perspective it is important to improve drug stability and also to release the drugs in a sustainable manner inside the cells. Development of nanoscale drug delivery system that allows a slow release of drug over prolonged periods of time is important to thus avoid burst effects. Our results show that Zn-RIF-Tf-QDs meet these criteria. We further show that Zn-RIF-Tf-QD selectively target of Zn-RIF into macrophages and dendritic cells, but not into A549 lung epithelial. In summary, we conclude that 1. Zn-RIF-Tf-QDs have a great potential as anti-TB molecules with reduced side effects, and 2. Transferrin-functionalized QDs can be used as a novel and efficient drug delivery system in TB therapy.

## Methods

### Chemicals, reagents and cell culture conditions

*M. smegmatis* mc^2^155 and *M. bovis*-BCG were grown in Middlebrook’s 7H9 broth medium supplemented with 1% OADC (*Oleic Albumin Dextrose Catalase*) and 0.05% Tween 80 (Merck) at 120 r.p.m. To stabilize GFP, medium was supplemented with hygromycin (50 μg/ml). The mouse macrophage RAW 264.7 cells, peritoneal macrophages and dendritic cells were cultured in DMEM Dulbecco’s modified Eagle’s medium (DMEM; HiMedia, Mumbai, India) supplemented with 10% fetal bovine serum, 1% penicillin-streptomycin solution, and 1% L-glutamine. FITC (Fluorescein isothiocyanate), rifampicin, zinc nitrate and sodium borohydritewere purchased from Sigma (St. Louis, USA). MTT [3-(4, 5-dimethylthiazol-2-yl)-2, 5-diphenyltetrazolium bromide] was purchased from MP Biomedicals (USA).

### Isolation of peritoneal macrophages and dendritic cells

Peritoneal macrophages and dendritic cells were isolated from 6 to 8 week old BALB/c mice. All mice were maintained in high efficiency particulate air (HEPA) filter bearing cages under 12 h light cycles in our animal facility, and were given sterile chow and autoclaved water *ad libitum*. All animal experiments were performed in accordance with national guidelines for the care and handling of laboratory animals and have been approved by the Institutional Animal Care and Use committee of KIIT University (Approval Number: KSBT/IAEC/2013/MEET1/A11). Peritoneal macrophages were isolated by following a previously published protocol^44^. Similarly, dendritic cells (DCs) were isolated as described previously^45^. Bone marrow progenitor cells were stimulated with 20 ng/ml of granulocyte/macrophage colony stimulating factor (GM-CSF) for DC proliferation and maturation.

### Synthesis of Zinc-Rifampicin complex and its encapsulation with transferrin coupled silver quantum dots

For the synthesis of Zn-RIF complex, 18 mg (0.095 mM) of hydrated zinc nitrate and 80 mg (0.097 mM) rifampicin were dissolved in methanol and stirred in a 50 ml round bottom flask for 3–4 h. The precipitate was collected, dried, weighed and dissolved in dH_2_O for further experiments. Zn-RIF complex was characterized by Fourier transform infrared spectroscopy (FTIR) (Nicolet iS5, Thermo Scientific, India), UV-Visible (Epoch, BioTek, Germany), X-ray photoelectron spectroscopy (XPS) (S/N: 10001, Prevac, Poland), Powder X-ray diffraction (XRD) (Shimatzu-6100, Japan) and Nuclear magnetic resonance (NMR) (AVANCE 400, Bruker, Switzerland).

Silver QDs were synthesized as described previously^35^. Briefly, silver QDs were synthesized by reduction of silver nitrate (1 mM) by addition of excess of ice cold sodium borohydride (2 mM) solution by vigorous stirring at room temperature. QDs were synthesized in less than a minute reaction time. The stoichiometric ratio of silver nitrate to sodium borohydride is very critical for the synthesis of QDs.

To synthesize Zn-RIF encapsulated transferrin conjugated silver QDs, 1 mg/ml of transferrin and 500 μg/ml of Zn-RIF complex were added together with 1 mM concentration of silver nitrate. Then the reaction mixture was reduced by excess ice cold sodium borohydrate solution.

### Characterization of Zn-RIF encapsulated transferrin conjugated QDs (Tf-QDs)

After encapsulation of Zn-RIF complex into transferrin conjugated QDs (Zn-RIF-Tf-QDs), the synthesized Zn-RIF-Tf-QDs conjugate was characterized by UV-Vis spectroscopy, FTIR analysis and transmission electron microscopy (TEM). For TEM, a drop of aqueous solution of Zn-RIF-Tf-QDs was placed on the carbon-coated copper grids. The samples were dried and kept overnight under a desiccator before loading them onto a specimen holder. TEM measurements were performed on JEM-2100, HRTEM, JEOL, JAPAN operating at 200 kV.

### *In vitro* killing assay

To determine the antimycobacterial activity of Zn-RIF and Zn-RIF-Tf-QDs complexes, 4–5 × 10^5^ bacteria were incubated with various concentrations of these complexes in 7H10 medium in 96-well round bottom plates in triplicates. Bacteria were harvested at the indicated time points and the number of colony forming units (CFUs) was assayed by plating suitably diluted cultures on 7H10 agar plates. All samples were plated in triplicate and values were averaged from three independent trials.

### Intracellular killing assay

RAW 264.7 macrophage cells were infected as described previously^16^. Briefly, RAW 264.7 cells (2 × 10^5^cells/well) were infected with *M. smegmatis* and *M. bovis-*BCG at a multiplication of infection (MOI) 10 for 2 h followed by the treatment with different concentrations of drug complexes. Extracellular bacteria were killed by addition of 20 μg/ml gentamycin. Cells infected with bacteria alone were used as control. After the incubation period, cells were washed, lysed with 0.5% triton-X-100 and intracellular survival was determined by plating serially diluted samples on 7H10 agar plates and the *M. smegmatis* and *M. bovis*-BCG colonies were enumerated after 3 days and 1 month, respectively.

### Cytotoxicity assay

RAW 264.7 cells (1 × 10^5^cells/well) were grown in 24-well plates for 24 h followed by treatment with different concentrations of drug complexes for another 24 h. Cell viability was determined by MTT assay as described previously^16^.

### Endocytosis of drug encapsulated QDs by macrophages, dendritic cells and lung epithelial cells

Zn-RIF-Tf-QD conjugate was labeled by addition of 5 μg/ml of FITC and the mixture was stirred continuously for 8 h in dark. RAW 264.7 cells, peritoneal macrophages, dendritic cells and lung epithelial cells (1 × 10^5^ cells/ml) were grown on glass cover slips in 24-well plate. FITC labeled Zn-RIF-Tf-QD conjugate were added to the cells. DMEM media with FITC only was taken as control. Then the cells were incubated for 1 h, washed with 1× PBS, fixed with 4% paraformaldehyde for 30 min at 37 °C and mounted on a glass slide. The cells were observed under a fluorescence microscope (Nikon, Japan).

### Intracellular stability of Zn-RIF-Tf-QD conjugate

Zn-RIF-Tf-QD conjugate were labeled as described above. RAW 264.7 cells, peritoneal macrophages and dendritic cells grown on glass cover slips were treated with FITC labeled Zn-RIF-Tf-QD conjugate for 48 h. After the incubation period, cells were washed, fixed and mounted by DAPI containing mounting solution. The images were visualized by using fluorescence microscopy.

### Immunofluoroscence studies

The immunofluorescence study was performed to evaluate the localization of Zn-RIF-Tf-QD conjugate in mouse macrophages. RAW 264.7 cell lines were grown on glass cover slips and treated with FITC labeled Zn-RIF-Tf-QD conjugate. After 3 h of incubation period, cells were fixed with 4% paraformaldehyde, permeabilized with 0.2% saponin and blocked with 5% BSA. Then the cells were incubated with 1:200 diluted rabbit LAMP-1 primary antibody ( Cell Signaling) for 1 h at room temperature, washed with 1× PBS and incubated with Alexa fluor conjugated secondary antibody (1:200) for 2 h. Then the cells were washed twice with 1× PBS and mounted with DAPI containing mounting solution (Invitrogen). Localization of FITC labeled Zn-RIF-Tf-QD conjugate complexes were visualized using the fluorescence microscope (Nikon, Japan).

### *In-vitro* cytokinesis-blocked micronucleus assay

Micronucleus assay measures the DNA damage resulting from exposure to toxic agents^46^. *In vitro* cytokinesis-blocked micronucleus assay (CBMN) was performed by treating the mouse macrophages with 1 and 10 μg/ml doses of both Zn-RIF and Zn-RIF-Tf-QD conjugatefor 24 h. Then the cells were treated with cytochalasin B (8 μg/ml) (MP-BIO) and were incubated for another 22 h. Cells were harvested, centrifuged and the pellet was fixed with fixative (3:1 methanol/acetic acid). The cells were kept at 4 °C for at least 4 days, added drop wise on glass slides and dried. The slides were then stained with propidium iodide (4 μg/ml) and analyzed by fluorescence microscopy to visualize binucleated and micronuclei forming cells.

### Comet assay

The alkaline comet assay was performed to check the effect of Zn-RIF-Tf-QD conjugate on DNA damage. Macrophages were treated with 1 and 10 μg/ml doses of both Zn-RIF and Zn-RIF-Tf-QD conjugatefor 24 h at 37 °C. Untreated cells were used as a control. Cell suspensions (400 μL) were mixed with 1% low melting point agarose (1 ml) and added to glass cavity slides (Blue Label Scientifics Pvt Ltd, Mumbai, India). The agarose was allowed to solidify and then the slides were submerged in lysis solution (1.2 M sodium chloride, 100 mM EDTA disodium salt, 0.1% SDS, 0.26 M NaOH [pH. 13]) for 2 h at 4 °C in the dark. After lysis, the slides were washed with dH_2_O, transferred to an electrophoresis unit, covered with freshly prepared electrophoresis buffer (0.03 M NaOH, 2 mM EDTA disodium salt, pH 12.3), left for unwinding of DNA for 30 minutes, and the cells were electrophoresed for 22 minutes at 22 V. The cells were neutralized with neutralization buffer (500 mMTrisHCl, pH 8.0) for 15 minutes, washed 3–4 times with dH_2_O, and stained with PI (4 μg/mL) for 1 hour. The slides were dried and then observed using the fluorescence microscope.

### Statistical analysis

Significant differences between the groups were determined by One-way and Two-way ANOVA. All the statistical calculations were performed with the help of GraphPad prism version 5.0. Significance was indicated as *** for P < 0.001; ** for P < 0.01 and * for P < 0.05.

## Additional Information

**How to cite this article**: Pati, R. *et al*. Encapsulation of zinc-rifampicin complex into transferrin-conjugated silver quantum-dots improves its antimycobacterial activity and stability and facilitates drug delivery into macrophages. *Sci. Rep.* **6**, 24184; doi: 10.1038/srep24184 (2016).
